# Systematic Lymph Node Dissection May Be Abolished in Patients With Apparent Early-Stage Low-Grade Mucinous and Endometrioid Epithelial Ovarian Cancer

**DOI:** 10.3389/fonc.2021.705720

**Published:** 2021-09-06

**Authors:** Jiayu Chen, Jie Yin, Yan Li, Yu Gu, Wei Wang, Ying Shan, Yong-Xue Wang, Meng Qin, Yan Cai, Ying Jin, Lingya Pan

**Affiliations:** Department of Obstetrics and Gynecology, Peking Union Medical College Hospital, Chinese Academy of Medical Sciences and Peking Union Medical College, Beijing, China

**Keywords:** epithelial ovarian cancer, low grade, lymph nodes, metastasis, lymph node dissection

## Abstract

**Objective:**

To investigate whether systematic lymph node dissection can confer clinical benefits in patients with apparent early-stage low-grade epithelial ovarian cancer.

**Methods:**

Patients with apparent early-stage low-grade epithelial ovarian cancer seen at Peking Union Medical College Hospital from January 1, 2005, to December 31, 2015, were retrospectively enrolled. Patients with other histological types and those who did not receive necessary adjuvant chemotherapy were excluded. Data collection and long-term follow-up were performed. According to the removed lymph node number, three groups based on surgical methods were used: abnormal lymph node resection, pelvic lymphadenectomy, and systematic lymph node dissection to control surgical quality. Their effects on prognosis were analyzed in pathological subgroups.

**Results:**

A total of 196 patients were enrolled; 30.1% of patients had serous, 42.3% of patients had mucinous, and 27.6% of patients had endometrioid carcinoma, of which 51 (26.0%), 96 (49.0), and 49 (25.0%) patients were treated with the above surgical methods, respectively. The occult lymph node metastasis rate was 14 (7.1%), and only five (2.6%) of apparent early-stage patients were upstaged due to lymph node metastasis alone. Systematic lymph node dissection did not benefit progression-free survival or disease-specific overall survival of apparent early-stage low-grade mucinous and endometrioid epithelial ovarian cancer but prolonged progression-free survival of apparent early-stage low-grade serous patients (OR, 0.231, 95% CI, 0.080, 0.668, p = 0.007).

**Conclusions:**

Systematic lymph node dissection may be abolished in patients with apparent early-stage low-grade mucinous and endometrioid epithelial ovarian cancer but may be considered for apparent early-stage low-grade serous patients.

## Introduction

Ovarian cancer is the most lethal tumor of all gynecological malignancies, approximately 90% of which are epithelial ovarian cancer (EOC) (1). Complete staging surgery and necessary adjuvant chemotherapy are the standard treatments for EOC patients according to the National Comprehensive Cancer Network (NCCN) guidelines (2). Systematic lymph node dissection (SLND) is an essential procedure that has been a part of complete staging procedures since 1988, including pelvic and para-aortic lymphadenectomy (2, 3). In early-stage EOC, SLND helps doctors acquire a sufficient number of lymph nodes (LNs) to identify occult LN metastases and guide adjuvant chemotherapy decisions by accurate staging (4, 5).

However, the low LN metastatic rate and upstaging rate in apparent early-stage low-grade EOC (LGEOC) reported in few studies challenge the necessity of SLND (6, 7). Nevertheless, those studies had intrinsic limitations: uncontrolled surgery quality, non-parallel prognostic factors, and partially missing clinical and prognostic data. As a result, the role of SLND in apparent early-stage LGEOC is still unclear. Low incidence increases the difficulty of studying LGEOC, but its unique features compared with high-grade EOC (HGEOC) have increased the urgency and necessity of studying its clinical characteristics and establishing an individualized treatment (8–11).

This study aims to determine the LN metastatic patterns of apparent early-stage LGEOC patients, including patients with low-grade serous ovarian cancer (LG-SOC), low-grade mucinous ovarian cancer (LG-MOC), and low-grade endometrioid ovarian cancer (LG-EOC), and to explore the survival benefit of SLND on them. The primary endpoint is disease-specific overall survival (OS), and the secondary endpoint is progression-free survival (PFS).

## Materials and Methods

### Patients

**Figure 1:**
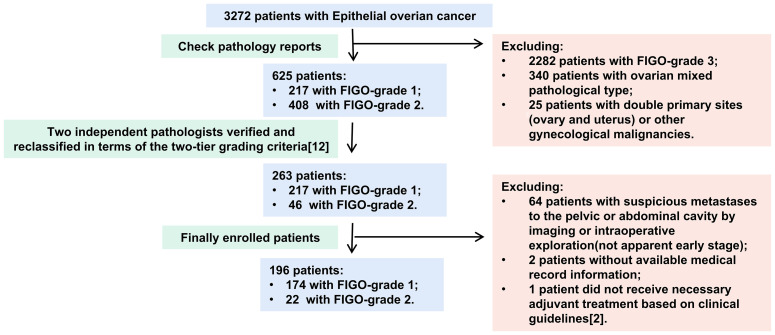
The flowchart of patients’ inclusion and exclusion.

The inclusion criteria were as follows: 1) diagnosed with LGEOC—LG-SOC, LG-MOC, or LG-EOC; 2) presented apparent early-stage disease; and 3) underwent staging surgery. Literature reported that all International Federation of Gynaecology and Obstetrics (FIGO) grade 1 and some FIGO grade 2 patients might belong to low grade according to the two-tier grading criteria (12), so two independent pathologists reclassified the primary lesion pathological sections of those patients into low and high grades in terms of the two-tier grading criteria (**Figure 1**). An apparent early stage was defined as a tumor localized to the bilateral adnexa (ovaries and fallopian tubes) and uterus on preoperative imaging and intraoperative exploration, similar to FIGO I–IIA stage (13). The exclusion criteria were as follows: 1) ovarian mixed pathology, double primary sites (ovary and uterus), and other gynecological malignancies; 2) no available medical record information; and 3) did not receive necessary adjuvant treatment based on clinical guidelines (2).

### Clinical Data Collection and Organization

This retrospective single-center study was conducted at the Department of Obstetrics and Gynecology, Peking Union Medical College Hospital, between January 1, 2005, and December 31, 2015, and approved by the Institutional Review Board. Medical history, surgical and pathological data, and postoperative treatment and follow-up data were collected continuously once a patient met the inclusion criteria and lacked the exclusion criteria. The general physical condition was assessed with the American Society of Anesthesiologists (ASA) classification (14). Pathologically explicit FIGO stages I to IIa were defined as early stage. The LN dissection methods were classified into three categories to control quality (5, 7, 15–19):

1. Group 1: no LN dissection or LN sampling—removal of none or a few LNs (less than ten pelvic LNs)
2. Group 2: pelvic lymphadenectomy—removal of more than 10 pelvic LNs
3. Group 3: SLND—removal of more than 10 pelvic LNs and five para-aortic LNs

All LN excision numbers were confirmed by pathology.

### Follow-Up

PFS was defined as the time between the date of diagnosis and the date of the first recurrence, the last follow-up, or death, whichever occurred first; while OS was the interval period from the date of diagnosis to the date of disease-specific death or last follow-up. Follow-up was conducted by consulting clinic records or telephone contact, and the cutoff date was between December 2020 and January 2021.

### Statistical Analysis

The measurement data were analyzed by ANOVA or a non-parametric test (Mann–Whitney U test), and the chi-square test was used to analyze hierarchical data. Patients lost to follow-up were excluded from the survival analysis. The reverse Kaplan–Meier method was used to calculate the median follow-up time; and 5- or 10-year PFS rates and OS rates were estimated according to the Kaplan–Meier curves. The log-rank test and Kaplan–Meier test were adopted as univariate analysis methods for identifying risk factors for PFS and OS, and those variables with p-values less than 0.2 were enrolled in the multivariate Cox regression analysis to identify independent risk factors. All p-values were two-sided, and differences were considered statistically significant with p ≤ 0.05 and when the 95% confidence interval (CI) did not cross 1. All statistical analyses were conducted with IBM SPSS Statistics 20 (IBM, Armonk, NY, USA).

## Results

### The Clinical Features of Low-Grade Epithelial Ovarian Cancer

In over 3,272 EOC patients, 263 (8.04%) were diagnosed with LGEOC (217 had FIGO-G1 disease and 46 had FIGO-G2 disease). One hundred ninety-six patients were eventually included in the study (**Figure 1**), of which 59 (30.1%) had LG-SOC, 83 (42.3%) had LG-MOC, and 54 (27.6%) had LG-EOC. Their clinical features are depicted in **Table 1**: more than half of patients were younger than 40 years at diagnosis and had a history of borderline ovarian tumor (BOT). The CA125 level varied remarkably, ranging from 0.32 to 65,065 U/ml. Recurrence occurred in 24.6% of patients, and disease-specific death occurred in 14.3% of patients. The 5-year survival rate was 88.0% (95% CI, 82.1%, 93.9%), and the 10-year survival rate was 74% (95% CI, 62.2%, 85.8%). Notably, 33 apparent early patients were classified as advanced patients due to postoperative pathology.

**Table 1:** Clinical information of apparent early-stage patients with different LN resection methods.

Mode of lymph node resection		Total n = 196	1 n = 51	2 n = 96	3 n = 49	p-Value
Age (years)	≤40	103 (52.6%)	28 (54.9%)	51 (53.1%)	24 (49.0%)	0.356
	40–60	76 (38.8%)	16 (31.4%)	37 (38.6%)	23 (46.9%)	
	>60	17 (8.6%)	7 (13.7%)	8 (8.3%)	2 (4.1%)	
Menopause	No	146 (74.5%)	35 (68.6%)	73 (76.0%)	38 (77.6%)	0.567
	Yes	50 (25.5%)	16 (31.4%)	23 (24.0%)	11 (22.4%)	
BMI		22.88 ± 3.76	22.67 ± 7.34	22.77 ± 3.72	22.31 ± 3.94	0.482
BOT history	No	97 (51.3%)	20 (51.3%)	47 (51.1%)	30 (62.5%)	0.129
	Yes	92 (48.6%)	19 (48.7%)	45 (48.9%)	18 (37.5%)	
ASA classification	I	110 (57.0%)	28 (56.0%)	58 (61.1%)	24 (50.0%)	0.159
	II	77 (39.9%)	18 (36.0%)	36 (37.9%)	23 (48.0%)	
	III	6 (3.1%)	4 (8.0%)	1 (1.0%)	1 (2.0%)	
CA125 level (U/ml)		66.3 (23.9, 227)	77.7 (37.3, 116)	49.1 (20.3, 228.5)	76.7 (28.8, 410)	0.243
Tumor size (cm)		10 (7, 15)	10 (7.75, 10)	10 (6, 15)	10 (7, 13)	0.752
Pathology	Serous	59 (30.1%)	22 (43.1%)	25 (26.0%)	12 (24.4%)	0.011
	mucinous	83 (42.3%)	18 (35.3%)	49 (51.0%)	16 (32.7%)	
	Endometrioid	54 (27.6%)	11 (21.6%)	22 (23.0%)	21 (42.9%)	
Tumor stage	Early	163 (83.2%)	38 (74.5%)	84 (87.5%)	41 (83.7%)	0.135
	Late	33 (16.8%)	13 (24.5%)	12 (12.5%)	8 (16.3%)	

BMI, body mass index; BOT, borderline tumor; ASA, American Society of Anesthesiologists; LN, lymph node.

### Different Lymph Node Dissection Modes and Lymph Node Metastasis Status

The three groups recruited 51, 96, and 49 patients, and all indexes but pathological type were balanced among them (**Table 1**). The number of LNs removed by different surgical methods and the LN metastatic status are described in **Table 2**. Fourteen patients (7.1%) had occult LN metastasis, including contralateral metastasis, bilateral metastasis, and skip metastasis that only had para-aortic LN metastasis and no pelvic LN metastasis. The most common metastatic site was iliac LNs (13/14, 92.9%), followed by para-aortic LNs (4/14, 28.6%), while only one patient had common iliac LN metastases (p < 0.001). LG-SOC had a significantly higher LN involvement rate than LG-MOC and LG-EOC (18.6% *vs.* 1.2% and 3.7%, p < 0.001).

**Table 2:** LN removed number, LN+ detection rate, and upstaging only due to LN metastasis among three LN dissection groups in all subgroups and pathological subgroups.

Mode of lymph node resection		1	2	3	p-Value
All N = 196	Number of pelvic LNs removed	0 (0, 2.5)	20 (16, 28)	25.5 (19.25, 30.75)	<0.001
	Number of para-aortic LNs removed	0 (0, 0)	0 (0, 2)	8 (6, 10)	<0.001
	Number of LN metastasis cases	4 (7.9%)	4 (4.2%)	6 (12.2%)	0.183
	Number of cases upstaging only due to LN metastasis	1 (2.0%)	1 (1.0%)	3 (6.1%)	0.154
Serous N = 59	Number of LN metastasis cases	3 (13.6%)	3 (12.0%)	5 (41.7%)	0.831
	Number of cases upstaging only due to LN metastasis	0 (0.0%)	1 (2.0%)	2 (16.7%)	0.150
Mucinous N = 83	Number of LN metastasis cases	0 (0.0%)	0 (0.0%)	1 (6.25%)	0.193
	Number of cases upstaging only due to LN metastasis	0 (0.0%)	0 (0.0%)	1 (6.25%)	0.193
Endometrioid N = 54	Number of LN metastasis cases	1 (9.1%)	1 (4.5%)	0 (0.0%)	0.677
	Number of cases upstaging only due to LN metastasis	1 (9.1%)	0 (0.0%)	0 (0.0%)	0.204

### The Effect of Lymph Node Dissection Mode on the Lymph Node Metastasis Detection and Upstaging Rates

Although a significantly higher number of pelvic and para-aortic LNs were removed from SLND than from other patients, no significant differences in the LN metastasis detection rate were observed between patients with any pathological subtype (**Table 2**). Moreover, only five (2.6%) patients were upstaged due to LN metastasis alone, and the rate of upstaging to stage IIIA1 was not affected by LN resection method (**Table 2**). Although the rate of upstaging in LG-SOC was higher than that in LG-MOC and LG-EOC, the differences were not statistically significant (5.1% *vs.* 1.2% and 1.9%, p = 0.452).

### The Effect of Lymph Node Dissection Mode on Survival

**Figure 2:**
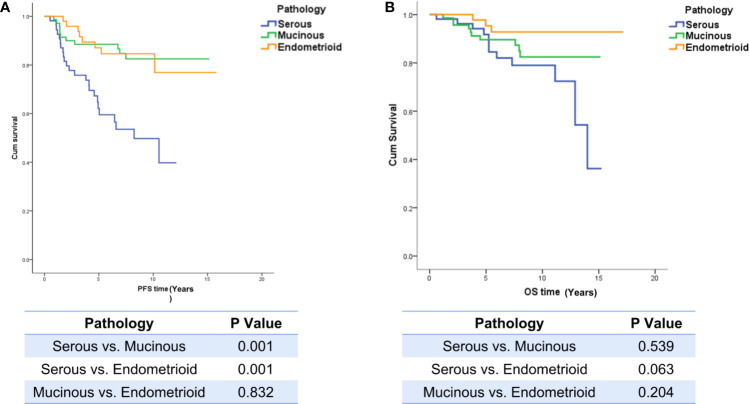
The influence of pathological types on PFS and OS. **(A)** The influence of pathological types on PFS. **(B)** The influence of pathological types on OS. The log-rank test was performed between any two pathological types, and a p-value of less than 0.05 was considered statistically significant. PFS, progression-free survival; OS, overall survival.

Overall, 10.7% of patients (21/196) were lost to follow-up and were excluded from the survival analysis, and the median follow-up time was 7.1 years (95% CI, 6.3, 7.5). The PFS of LG-SOC patients was significantly shorter than that of LG-MOC and LG-EOC patients, but there was no significant difference in OS among the groups: the 5-year survival rate was 82%, 89%, and 93% for LG-SOC, LG-MOC, and LG-EOC, respectively (**Figure 2**).

Considering that the population distribution of the three surgical groups was significantly different in terms of pathological type, we divided patients into pathological subgroups for prognosis analysis. To balance prognostic risk factors, univariate analysis was performed first (**Appendixes 1–3**), followed by Cox multivariate regression analysis (**Appendix 4**).

**Figure 3:**
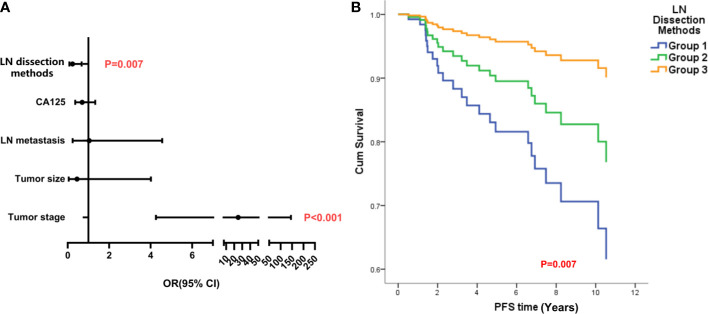
The analysis of independent risk factors on PFS of LG-SOC. **(A)** The forest figure of Cox multiple regression for PFS of LG-SOC, including items with p-values less than 0.3 in univariate analysis. Any item in which a p-value was less than 0.05 and the 95% CI for OR did not cross 1 was considered statistically significant. The p-value of the multivariate regression model was less than 0.001. **(B)** The survival curves of LN dissection methods on PFS after controlling other variables by the Cox test. OR, odds ratio; 95% CI, 95% confidence interval of OR; PFS, progression-free survival; LG-SOC, low-grade serous ovarian cancer; LN, lymph node.

In LG-SOC patients, the CA125 level, mode of LN resection, tumor size, tumor stage, and LN metastasis were considered in the Cox regression analysis of PFS; and mode of LN resection (odds ratio (OR), 0.231, 95% CI, 0.080, 0.668, p = 0.007) and tumor stage were identified as statistically significant factors. Lower number of LN retrieved and late stage were independent risk factors for PFS (**Figure 3**). The mode of LN resection was not considered in the Cox regression analysis of OS, and only higher tumor stage was an independent risk factor (**Appendixes 1, 4**).

In LG-MOC, the multiple-factor analysis identified BMI, mode of LN resection, tumor stage, and age as factors affecting OS and PFS (**Appendix 2**). LN dissection methods did not affect survival (PFS: OR, 0.530, 95% CI, 0.155, 1.811, p = 0.311; OS: OR, 0.684, 95% CI, 0.173, 2.694, p = 0.587), while tumor stage was the only risk factor affecting both PFS and OS (**Appendix 4**).

In LG-EOC, not enough items could be considered in the Cox regression analysis of PFS; the p-value of the log-rank test for LN resection mode was 0.059 (**Appendix 3**). Age, mode of LN resection, and tumor size were considered in the Cox regression analysis of OS, but we failed to find any significant risk factors (**Appendixes 3, 4**).

### The Effect of Lymph Node Dissection Mode on Operation-Related Complications

The operative time, blood loss, perioperative complication incidence, and incidence of lymphocysts significantly increased as the number of LNs removed increased (**Table 3**).

**Table 3:** Comparison of operative time, blood loss, blood transfusion, and perioperative complications among different lymph node resection methods.

Mode of lymph node resection	1 N = 51	2 N = 96	3 N = 49	p-Value
Operative time (min)	190.3 ± 84.9	213.1 ± 53.5	251.1 ± 38.2	0.001
Blood loss (ml)	300 (137.5, 600)	300 (200, 400)	400 (300, 500)	0.001
Blood transfusion	8 (15.7%)	15 (15.6%)	5 (10.2%)	0.671
Perioperative complication	7 (13.7%)	19 (19.8%)	17 (34.7%)	0.031
Lymphatic cyst	1 (2.0%)	16 (16.7%)	13 (26.5%)	0.002

## Discussion

As a rare form of ovarian cancer, LGEOC has a low incidence, accounting for approximately 6%–8% of EOC cases (8–11), and has unique clinical features as compared with HGEOC: low onset age, a history of BOT, an increased proportion of early-stage patients, a low LN metastasis rate, and a favorable prognosis (5, 7–11, 18, 20–25).

Since neither preoperative imaging nor intraoperative LN observation can predict LN metastasis precisely, 20%–30% of apparent early-stage EOC patients have LN metastasis (26–29). As a result, the aim of SLND in apparent early-stage EOC is to find occult LN metastasis and guide surgical–pathological staging (4, 5). The patients who experience upstaging receive adjuvant chemotherapy, which may benefit the prognosis.

However, the significantly lower incidences of LN metastasis and upstaging in LGEOC than in HGEOC challenge the necessity of SLND in apparent early-stage patients. The LN involvement rate of LGEOC was 2.9% (7). Similarly, Nasioudis et al. (25) recognized that the LN metastasis rates of LG-SOC, LG-MOC, and LG-EOC patients were significantly lower than those of high-grade patients (9.0% *vs.* 14.4% and 1.7% *vs.* 5.1% and 1.7% *vs.* 8.6%, respectively). Moreover, Minig et al. (6) observed that only 2.4% of apparent stage I LGEOC was upstaged by LN involvement alone. A meta-analysis of retrospective studies reported that the proportion was only 2.9% (7). In our study, the LN involvement rate was only 7.1%, and only 2.6% of apparent early-stage LGEOC patients were upstaged by LN involvement alone. Although SLND significantly increased the LN involvement rate among early stage EOC in one randomized controlled trial (RCT) research (18), the LN dissection methods did not affect the LN+ detection rate or upstaging rate in this study. This may be due to the low rate of LN metastasis in LGEOC, considering more than half of patients included in Maggioni’s study were FIGO stage 3, and 60 patients were diagnosed as clear-cell, undifferentiated, and other pathology types. Given these findings, we believe that upstaging should not be the reason for performing SLND in apparent early-stage LGEOC patients. Notably, LG-SOC had a higher LN+ rate and upstaging rate than the other two pathological types.

Prolonging survival is the other reason for SLND, based on the hypothesis that dissection of chemotherapy-resistant metastatic LNs could improve patient prognosis (referred to as the chemotherapeutic drug sanctuary hypothesis) (30). In a multicenter retrospective study including 639 patients with apparent early-stage EOC, researchers found that pelvic and para-aortic lymphadenectomy improves disease-free survival but not OS (31). However, proof of a survival benefit of SLND in apparent early-stage LGEOC patients is still lacking. In this paper, SLND did not prolong PFS or OS among LG-EOC and LG-MOC patients, but it did significantly prolong PFS in LG-SOC patients. LGEOC patients diagnosed at younger age have longer survival and may experience multiple recurrences, so a shorter PFS means those patients may need to undergo multiline treatment in a longer time, resulting in a significant decrease in quality of life and an increase in financial burden. Although the European Society for Medical Oncology–European Society of Gynaecological Oncology (ESMO–ESGO) consensus conference recommends that SLND may be questioned in some histological subtypes (LG-SOC or mucinous carcinoma of expansile subtype) due to a low prevalence of LN metastases (33), we insist that LG-SOC patients may still need SLND in terms of PFS benefit.

Another concern of performing SLND in LGEOC patients is that SLND is a complicated surgery, so even experienced gynecological oncologists encounter various complications (7). A study reported that 26.9% of patients with SLND experienced perioperative complications, and 54.7% had postoperative complications (32). Therefore, it is necessary to balance the benefits with the risks. We observed that the operative time, blood loss, perioperative complications, and lymphocyst count were significantly increased with an increase in the LN removal scope.

This retrospective study has inherent limitations. We could not control or include all prognostic factors. In addition, the information collection had some deficiencies, such as insufficient details of LN metastatic sites, possible omissions regarding surgical complications, and incomplete Immunohistochemistry (IHC) information, making it impossible to analyze correlated issues.

## Conclusion

In conclusion, SLND may be abolished in patients with apparent early-stage LG-MOC and LG-EOC since it did not significantly improve patient staging or prognosis or increase surgery risk. Patients with apparent LG-SOC may still need SLND, considering its prolongation of PFS.

## Data Availability Statement

The original contributions presented in the study are included in the article/**Supplementary Material**. Further inquiries can be directed to the corresponding authors.

## Ethics Statement

Written informed consent was obtained from the individual(s) for the publication of any potentially identifiable images or data included in this article.

## Author Contributions

All authors contributed to the study conception and design and material preparation. The first draft of the manuscript was written by JC, and all authors commented on previous versions of the manuscript. All authors contributed to the article and approved the submitted version.

## Funding

This project was supported by CAMS Innovation Fund for Medical Sciences (CIFMS-2017-I2M-1-002) and The Fund of The National Key R&D Program of China 2016YFC1303700 (Affiliated project 2016YFC1303701).

## Conflict of Interest

The authors declare that the research was conducted in the absence of any commercial or financial relationships that could be construed as a potential conflict of interest.

## Publisher’s Note

All claims expressed in this article are solely those of the authors and do not necessarily represent those of their affiliated organizations, or those of the publisher, the editors and the reviewers. Any product that may be evaluated in this article, or claim that may be made by its manufacturer, is not guaranteed or endorsed by the publisher.
