# Supplementary material for: Systematic Lymph Node Dissection May Be Abolished in Patients With Apparent Early-Stage Low-Grade Mucinous and Endometrioid Epithelial Ovarian Cancer
Source: Front Oncol. 2021 Sep 6;11:705720. doi: 10.3389/fonc.2021.705720 (PMC8450513; doi:10.3389/fonc.2021.705720)
Supplement: Supplementary file 1 [file DataSheet_1.doc]

**Appendix 1**. Univariate analysis of progression-free survival(PFS) and overall survival(OS) for LG-SOC.

|  | | No recurrence  (n=35) | Recurrence  (n=24) | Univariate analysis  (P value) | Survival  (n=42) | Death  (n=12) | Univariate analysis  (P value) |
| --- | --- | --- | --- | --- | --- | --- | --- |
| Age(years) | ≤40 | 18 | 11 | 0.738 | 24 | 5 | 0.780 |
|  | 40-60 | 15 | 11 |  | 30 | 6 |  |
|  | >60 | 2 | 2 |  | 3 | 1 |  |
| BMI | <24 | 19 | 14 | 0.897 | 26 | 7 | 0.441 |
|  | ≥24 | 67 | 6 |  | 10 | 3 |  |
| ASA classification | I | 21 | 11 | 0.514 | 26 | 6 | 0.292 |
|  | II | 12 | 10 |  | 16 | 6 |  |
|  | III | 2 | 1 |  | 3 | 0 |  |
| CA125 level(U/ml) | <35 | 8 | 2 | 0.038 | 10 | 0 | 0.077 |
|  | 35-200 | 8 | 12 |  | 13 | 7 |  |
|  | 200-500 | 6 | 2 |  | 8 | 0 |  |
|  | ≥500-1000 | 12 | 2 |  | 3 | 1 |  |
| Mode of lymph node resection^*^ | 1 | 9 | 13 | 0.006 | 16 | 6 | 0.524 |
|  | 2 | 16 | 9 |  | 21 | 4 |  |
|  | 3 | 10 | 2 |  | 10 | 2 |  |
| Tumor size(cm) | ≤10 | 22 | 15 | 0.097 | 28 | 9 | 0.049 |
|  | 10 | 8 | 2 |  | 10 | 0 |  |
| Tumor stage | Early-stage | 28 | 9 | <0.001 | 34 | 3 | 0.006 |
|  | Late-stage | 7 | 15 |  | 13 | 9 |  |
| Lymph node metastasis | Yes | 5 | 6 | 0.089 | 5 | 6 | 0.002 |
|  | No | 26 | 14 |  | 34 | 6 |  |

^*^1-Abnormal LN resection ; 2-pelvic lymphadenectomy; 3-SLND.

BMI-Body mass index, BOT-borderline tumor, ASA-American Society of Anesthesiologists.

**Appendix 2**. Univariate analysis of progression-free survival(PFS) and overall survival(OS) for LG-MOC.

|  | | No recurrence  (n=72) | Recurrence  (n=11) | Univariate analysis  (P value) | Survival  (n=73) | Death  (n=10) | Univariate analysis  (P value) |
| --- | --- | --- | --- | --- | --- | --- | --- |
| Age(years) | ≤40 | 44 | 7 | 0.547 | 44 | 7 | 0.251 |
|  | 40-60 | 21 | 2 |  | 22 | 1 |  |
|  | >60 | 7 | 2 |  | 7 | 2 |  |
| BMI | <24 | 33 | 6 | 0.157 | 34 | 5 | 0.236 |
|  | ≥24 | 24 | 1 |  | 24 | 1 |  |
| ASA classification | I | 41 | 8 | 0.693 | 41 | 8 | 0.496 |
|  | II | 27 | 3 |  | 28 | 2 |  |
|  | III | 3 | 0 |  | 3 | 0 |  |
| CA125 level(U/ml) | <35 | 37 | 5 | 0.719 | 37 | 5 | 0.446 |
|  | 35-200 | 24 | 2 |  | 25 | 1 |  |
|  | 200-500 | 4 | 1 |  | 4 | 1 |  |
|  | ≥500 | 5 | 1 |  | 5 | 1 |  |
| Mode of lymph node resection^*^ | 1 | 15 | 3 | 0.193 | 16 | 2 | 0.227 |
|  | 2 | 41 | 8 |  | 41 | 8 |  |
|  | 3 | 16 | 0 |  | 16 | 0 |  |
| Tumor size(cm) | ≤10 | 28 | 5 | 0.983 | 28 | 4 | 0.717 |
|  | 10 | 41 | 4 |  | 42 | 4 |  |
| Tumor stage | Early-stage | 67 | 8 | 0.005 | 68 | 7 | 0.001 |
|  | Late-stage | 5 | 3 |  | 5 | 3 |  |
| Lymph node metastasis | Yes | 1 | 0 | 0.659 | 1 | 0 | 0.645 |
|  | No | 71 | 11 |  | 72 | 10 |  |

^*^1-Abnormal LN resection ; 2-pelvic lymphadenectomy; 3-SLND.

BMI-Body mass index, BOT-borderline tumor, ASA-American Society of Anesthesiologists.

**Appendix 3**. Univariate analysis of progression-free survival(PFS) and overall survival(OS) for LG-EOC.

|  | | No recurrence  (n=46) | Recurrence  (n=8) | Univariate analysis  (P value) | Survival  (n=51) | Death  (n=3) | Univariate analysis  (P value) |
| --- | --- | --- | --- | --- | --- | --- | --- |
| Age(years) | ≤40 | 19 | 4 | 0.605 | 22 | 1 | 0.225 |
|  | 40-60 | 24 | 3 |  | 26 | 1 |  |
|  | >60 | 3 | 1 |  | 3 | 1 |  |
| BMI | <24 | 4 | 24 | 0.884 | 26 | 2 | 0.927 |
|  | ≥24 | 2 | 14 |  | 15 | 1 |  |
| ASA classification | I | 24 | 5 | 0.876 | 28 | 1 | 0.357 |
|  | II | 22 | 3 |  | 23 | 2 |  |
|  | III | 0 | 0 |  | 0 | 0 |  |
| CA125 level(U/ml) | <35 | 10 | 0 | 0.759 | 10 | 0 | 0.476 |
|  | 35-200 | 19 | 3 |  | 21 | 1 |  |
|  | 200-500 | 8 | 1 |  | 8 | 1 |  |
|  | ≥500 | 8 | 1 |  | 9 | 0 |  |
| Mode of lymph node resection^*^ | 1 | 4 | 7 | 0.059 | 9 | 2 | 0.201 |
|  | 2 | 1 | 21 |  | 22 | 0 |  |
|  | 3 | 3 | 18 |  | 20 | 1 |  |
| Tumor size(cm) | ≤10 | 32 | 5 | 0.444 | 34 | 3 | 0.275 |
|  | 10 | 10 | 3 |  | 14 | 0 |  |
| Tumor stage | Early-stage | 44 | 7 | 0.415 | 48 | 3 | 0.625 |
|  | Late-stage | 2 | 1 |  | 3 | 0 |  |
| Lymph node metastasis | Yes | 2 | 0 | 0.608 | 2 | 0 | 0.736 |
|  | No | 44 | 4 |  | 49 | 3 |  |

^*^1-Abnormal LN resection ; 2-pelvic lymphadenectomy; 3-SLND.

BMI-Body mass index, BOT-borderline tumor, ASA-American Society of Anesthesiologists.

**Appendix 4**. Cox multivariate analysis of progression-free survival(PFS) and overall survival(OS).

|  | PFS | | | | OS | | | |
| --- | --- | --- | --- | --- | --- | --- | --- | --- |
| Pathology | Item | P value | Exp(B) | 95% CI | Item | P value | Exp(B) | 95% CI |
| LG-SOC | CA125 | 0.271 | 0.694 | 0.362, 1.330 | CA125 | 0.342 | 0.608 | 0.218, 1.696 |
|  | LN dissection methods | 0.007 | 0.231 | 0.080, 0.668 | Tumor size | 0.979 | - | - |
|  | Tumor size | 0.473 | 0.449 | 0.050, 4.008 | Tumor stage | 0.241 | 5.437 | 0.319, 93.787 |
|  | Tumor stage | <0.001 | 24.835 | 4.253, 145.029 | LN metastasis | 0.320 | 3.021 | 0.342, 26.696 |
|  | LN metastasis | 0.962 | 1.036 | 0.236, 4.555 | - | - | - | - |
| LG-MOC | BMI | 0.143 | 0.198 | 0.023, 1.728 | Age | 0.589 | 1.395 | 0.418, 4.658 |
|  | LN dissection methods | 0.311 | 0.530 | 0.155, 1.811 | BMI | 0.118 | 0.116 | 0.008, 1.726 |
|  | Tumor stage | 0.003 | 15.026 | 2.584, 87.366 | LN dissection methods | 0.587 | 0.684 | 0.173, 2.694 |
|  | - | - | - | - | Tumor stage | 0.001 | 59.893 | 5.298, 679.104 |
| LG-EOC | - | - | - | - | Age | 0.288 | 2.564 | 0.451, 14.567 |
|  | - | - | - | - | LN dissection methods | 0.358 | 0.463 | 0.090, 2.393 |
|  | - | - | - | - | Tumor size | 0.552 | 0.923 | 0.708, 1.202 |
